# Aptitude and experience as predictors of grammatical proficiency in adult Greek-English bilinguals

**DOI:** 10.3389/fpsyg.2022.1062821

**Published:** 2022-12-20

**Authors:** Leonarda Prela, Miquel Llompart, Ewa Dąbrowska

**Affiliations:** ^1^Chair of Language and Cognition, Department of English and American Studies, Friedrich-Alexander-Universität Erlangen-Nürnberg, Erlangen, Germany; ^2^Department of Translation and Language Sciences, Universitat Pompeu Fabra, Barcelona, Spain; ^3^Department of English Language and Linguistics, University of Birmingham, Birmingham, United Kingdom

**Keywords:** language aptitude, grammar, grammatical proficiency, bilingualism, ultimate attainment, individual differences, grammatical sensitivity, second language acquisition

## Abstract

It has been shown that individuals exhibit great variability in second language (L2) ultimate attainment. Some speakers reach native-like proficiency, others only achieve a rudimentary command and many lie in the middle. Individual differences research has partly attributed different degrees of L2 attainment to (language) aptitude. Initially considered irrelevant for first language (L1) acquisition, aptitude was viewed as a compensatory ability for adults’ *disadvantage* in L2 learning. In this line of thought, adults and children are viewed as fundamentally different and rely on different language learning mechanisms. However, aptitude might not be so irrelevant for the L1. Together with input the two factors are found to account for individual differences not only in L2 but also L1 development. Recent research has specifically shown that native grammatical attainment may be modulated by aptitude and input. In this respect, the aim of the current study is to examine the effects of these two predictors (namely input and aptitude) on both L1 and L2 grammatical attainment in the same speakers. Our participants (*N* = 75) were all native speakers of Greek who learned English as a foreign language in their home country and immigrated to the United Kingdom in adulthood (*mean age of arrival* = 27.3, *SD* = 6.4). Grammatical proficiency was measured through a grammaticality judgement task administered in both the L1 and the L2. Aptitude was measured through the Sentence Pairs task (based on the Words In Sentences test from the MLAT battery). Amount of input was measured using the traditional measure, length of residence (LoR) and a new cumulative measure that spanned across the participants’ life. The two measures were pitted against each other in the analysis. We found robust effects of aptitude in both the L1 and the L2, with the effect being even stronger for the L1. As expected, our new cumulative measure of exposure proved to be a better predictor of individual differences in grammatical proficiency. Last but not least, the effects of input were larger for the L2 than the L1.

## Introduction

It is widely accepted that adult second/foreign language (L2) learners rarely achieve nativelike proficiency in their L2 and that younger children are generally more successful in L2 learning than older children or adults (Flege et al., 1999; DeKeyser, 2012, 2000; Granena and Long, 2013). This is often attributed to a critical period or, more broadly, age constraints on language acquisition (see Lenneberg, 1967; Johnson and Newport, 1989; DeKeyser, 2000; Abrahamsson and Hyltenstam, 2009; DeKeyser et al., 2010; Granena and Long, 2013; Han, 2013). According to the Critical Period Hypothesis, adults and children are not equipotential when it comes to language learning: specifically, one or more of the learning mechanisms available to children is no longer available to mature learners. Following from this, it has been proposed that there is a “fundamental difference” in the way that children and adults acquire language, as well as the final outcome, with children exhibiting uniform success in contrast to adults who show great variability in ultimate attainment (Bley-Vroman, 1989; DeKeyser, 2000; Han, 2013).

This individual variation in adults has been the focus of individual differences research according to which there might be different factors responsible for (non)nativelike ultimate attainment which may or may not be confounded with age of acquisition (Muñoz and Singleton, 2011; Paradis, 2019; Li et al., 2022). This would help explain why there are cases in which early L2 onset does not seem to suffice for L2 nativelike proficiency (McDonald, 2000; Soto-Corominas et al., 2020). Among others, the factors behind this could either be external, like input (Flege et al., 1999; Flege, 2018) or learner-internal, for example language aptitude (Abrahamsson and Hyltenstam, 2008; Sparks et al., 2011; Li, 2015).

Building on the aforementioned research on individual differences, the current study aims to investigate the predictive power of input and aptitude over both the first (L1) and second language (L2) grammar of Greek-English adult bilinguals. By *input* we refer to the language experience that learners accumulated throughout their lives and up to the moment of testing; in this paper, the terms input and exposure will be used interchangeably. In L2 research, the term *aptitude* is usually defined as “an individual’s initial state of readiness and capacity for learning a foreign language, and probable facility in doing so given the presence of motivation and opportunity” (Carroll, 1981, p. 81). However, given some recent research suggesting that the abilities referred to as “aptitude” are also relevant for L1 acquisition and processing, we will use the term more broadly to refer to the capacity for learning language in general, i.e., both the L1 and the L2.

According to the original formulation of the Fundamental Difference Hypothesis (FDH; Bley-Vroman, 1989), learning a language in adulthood is different from child language acquisition because adults no longer have (full) access to Universal Grammar. More recently, the hypothesis has been reformulated in terms of explicit and implicit learning mechanisms: Children acquire the grammatical rules of their language effortlessly and without any conscious effort by relying on (possibly language specific) implicit learning mechanisms (Ullman, 2001; DeKeyser and Larson-Hall, 2005; Montrul, 2008; Kidd, 2012; Kidd and Arciuli, 2016), whereas adults need to rely, at least partly, on domain general explicit learning mechanisms. This is because implicit learning abilities are thought to decrease as a function of age (Ullman, 2001; Paradis, 2011; Granena and Long, 2013); because of this, adults have to resort to explicit learning strategies to compensate (Norris and Ortega, 2001; DeKeyser et al., 2010; Spada and Tomita, 2010). However, this distinction between adults and children has been questioned in two ways.

Firstly, there is not sufficient evidence showing that adults’ implicit skills are indeed worse than those of children. Adults certainly are capable of acquiring new knowledge implicitly, as demonstrated by a number of studies on (semi) artificial language learning (Marsden et al., 2013; Rebuschat, 2013; Grey et al., 2014). Whether these abilities are inferior to those of children is unclear. While a number of studies (e.g., Howard and Howard, 2013) report a decline in implicit learning as a function of age, most of these studies compared younger and older adults and hence do not speak to the question whether children are better implicit learners than adults. There are very few studies which compared the implicit learning abilities of young adults and children, and most of these found that implicit learning abilities continue to improve throughout childhood and adolescence and begin to decline only in later adulthood (see, e.g., Thomas et al., 2004; Lukács and Kemény, 2015).^1^

Secondly, research on ultimate L2 attainment comparing children and adults is inconclusive. Although Critical Period (CP) effects are reported in many studies (Asher and García, 1969; Ramsey and Wright, 1974; Johnson and Newport, 1989; Hyltenstam, 1992; Flege et al., 1999; Morford and Mayberry, 2000; Granena and Long, 2013), in some studies adults have been found to outperform younger learners in L2 learning (Asher and Price, 1967; Cenoz, 2003). This opposite pattern has been attributed to the fact that ‘the younger the better’ position might be domain-specific, with research supporting that this advantage is mainly associated with phonetic/phonological performance (Kirch, 1956; Flege et al., 1995, 1999) whereas grammar is not necessarily influenced by the same maturational constraints (Snow and Hoefnagel-Höhle, 1978). Indeed, the results of studies which investigated grammar are mixed: while some studies report a (near-)categorical difference between younger and older learners (Johnson and Newport, 1989; DeKeyser, 2000; DeKeyser et al., 2010; Abrahamsson, 2012), others (e.g., Sasaki, 1996; Street, 2017; Dąbrowska, 2019; Dąbrowska et al., 2020) have found considerable overlap between groups. These differences appear to be partly attributable to which aspects of grammar are investigated (L2 speakers do relatively well compared to native speakers on aspects of grammar which make a relatively clear contribution to meaning, in contrast to performance on grammatical distinctions which are largely redundant from a semantic point of view) and partly to the composition of the native control group (there is much more overlap between groups in studies in which the native control group was demographically more diverse – see Dąbrowska et al., 2020).

It is well known that some learners acquire a second language much faster than others. Such differences in speed of learning are attributed to individual differences in language aptitude, which, it has been argued, can sometimes compensate for age-related disadvantages in ultimate attainment. Foreign language aptitude involves a number of distinct abilities, including phonetic coding ability (the ability to discriminate and identify new language sounds), associative memory (the ability to learn a large number of items in a short time), grammatical sensitivity (awareness of the grammatical function of the different elements constituting a sentence) and inductive language learning ability, or the ability to identify grammatical/meaning patterns in an unknown language sample (Carroll, 1964, 1990). The last two abilities are the most relevant for the acquisition of grammar (Li, 2015).

Language aptitude tests such as the MLAT (Modern Language Aptitude Test, Carroll and Sapon, 1959) or PLAB (Pimsleur Language Aptitude Battery, Pimsleur, 1966; Pimsleur, 2004) were originally developed to predict the learning outcomes of the early stages of L2 learning in instructional settings. However, later research has also applied them to later stages of acquisition and ultimate attainment and to naturalistic settings (DeKeyser, 2000; Abrahamsson and Hyltenstam, 2008; Granena and Long, 2013). In fact, it has been argued that aptitude may be even more relevant in such settings because naturalistic learners must deduce the regularities and patterns of the language on their own without explicit instruction (Skehan, 1991; Granena, 2014). It is, indeed, in immersive settings where aptitude has yielded more intriguing but also conflicting results. Researchers concerned with this learning environment have mainly focused on the relationship between age and aptitude as a key point of investigation for the validity of the FDH.

More specifically, DeKeyser (2000) conducted a study with L1 Hungarian speakers in the United States in order to test the FDH. The participants were administered a grammaticality judgement task in English and an aptitude test in Hungarian. The results revealed that there was a correlation between aptitude and grammatical proficiency for the late arrivals, i.e., adult arrivals (0.33), but no correlation for the younger group (0.07). One possible explanation for this is that, when asked about which language they felt more comfortable with, the majority of the participants from the younger group in DeKeyser’s study reported English instead of Hungarian. This could have had affected their performance in the aptitude task (administered in Hungarian), which could in turn explain the lack of an effect of aptitude in the younger group.

By contrast, Abrahamsson and Hyltenstam (2008) tested 42 L2 speakers of Swedish with L1 Spanish exploring the relationship between aptitude and L2 proficiency among near-native L2 speakers. They found a striking correlation as high as 0.70 between aptitude and grammatical proficiency and what was even more surprising is that they found a relationship for the younger (age of onset <12) instead of the older (age of onset >12) group. In this study, the lack of an effect for the older group was attributed to the small sample size (n = 11 participants) as well as the narrow distribution of scores in the aptitude task for the same group. Finally, Granena and Long (2013) compared three groups of participants dividing them based on their age of onset (i.e., 3–6, 7–15, and 16–29 years) and did not find any relationship between morphosyntactic proficiency and aptitude in any of the three groups.

To add to this complex relationship, several studies have reported a relationship between aptitude and performance in the *native* language. Dąbrowska (2018) found a moderately strong correlation (0.46) between grammatical comprehension and (foreign) language aptitude as measured by the Language Analysis subtest from the PLAB in adult monolinguals. A more recent study by Llompart and Dąbrowska (in press) reports similar findings. In this last study, the researchers employed two different tasks (Language Analysis and Sentence Pairs, a test modelled on the Words in Sentences task of the MLAT) to measure aptitude, and an auditory grammaticality judgement task and a picture selection task to assess grammar. In the Language Analysis task, participants are required to deduce the meaning of a number of sentences in a foreign language by using the English translations of the words that form up those sentences. In the Sentence Pairs task, participants are presented with pairs of sentences, and they have to find the word of the second sentence that has the same function as a word in the first sentence which appears in capital letters. In line with Dąbrowska (2018), Llompart and Dąbrowska also report correlations over 0.40 between (foreign) language aptitude and native grammatical proficiency. Two other studies (Skehan and Ducroquet, 1988; Sparks et al., 2009) also found robust correlations between language aptitude measures (assessed when participants were in their teens) and earlier measures of L1 acquisition.

These findings are of interest because they challenge the view that child and adult language acquisition depend on distinct and fundamentally different systems, namely implicit and explicit learning mechanisms. If the existence of correlations between (explicit) language aptitude and attainment indicates that the latter relies on explicit learning, these findings suggest that explicit learning mechanisms may also be involved in L1 development (Dąbrowska, 2010; Llompart and Dąbrowska, 2020).

While the failure of most adult L2 learners to achieve nativelike proficiency is often attributed to maturational factors, other factors may also play a role. Among these, another important candidate is input (Huttenlocher, 1998; Huttenlocher et al., 2002; Clegg and Ginsborg, 2006; Hoff, 2006), which has been shown to be relevant for both L1 and L2 acquisition (Flege and Liu, 2001; Singleton and Ryan, 2004; Moyer, 2011). Furthermore, in connection with the current study, there is considerable evidence that variation in grammatical development can be explained to a great extent by differences in input (Gathercole, 2002; Montrul and Potowski, 2007; Chondrogianni and Marinis, 2011; Unsworth et al., 2014; Kaltsa et al., 2020).

It is clear that L1 learners as well as early L2 learners are more advantaged compared to late L2 learners in the amounts of input that they receive. Dąbrowska (2015) estimated that children receive at least 11,680 h of exposure to their L1 during the first years of their life (1–5 years old). Additionally, it has been shown that the benefits of exposure do not stop at a very young age but continue until adolescent years (Nippold, 1998; Berman, 2004, 2007; Nippold et al., 2005; Kaplan and Berman, 2015), and a recent large-scale study by Hartshorne et al. (2018) found that grammatical proficiency in native speakers continues to improve until about the age of 30. L2 learners, in contrast, not only receive less input overall (because they start learning later and typically continue to use the L1 alongside the L2), but also tend to get input of a poorer quality. Whether in instructional or immersive settings, L2 learners (in contrast to L1 learners) are often surrounded by other foreign language speakers and even their teachers may often be non-native speakers of the target language.

Traditionally, studies investigating the role of input have used length of residence (LoR) as a measure for the amount of input that speakers have received (Flege and Liu, 2001; Babcock et al., 2012; Saito and Brajot, 2013; Higby and Obler, 2016). However, as argued by Flege (2008), the use of LoR as an estimate of exposure assumes that exposure to the target language begins with arrival in the host country, and that immigrants with the same length of residence have received similar amounts of L2 input. Both of these assumptions are problematic. Many immigrants start learning or are exposed to the L2 before their arrival to the host country. Furthermore, individuals with the same LoR often differ tremendously in use of the target language: while some immigrants fully immerse themselves in the host environment from the beginning, others form closer bonds with their own communities, and may thus receive very little L2 input (Birdsong, 2009).

An additional methodological issue is the ‘age-length-onset’ problem (Stevens, 2006). In particular, Higby and Obler (2016) have argued that confounding issues between the three variables (age at testing, LoR and age of onset) give us a distorted picture of the exact contribution of each of these variables in L2 performance, making thus the relationship between aptitude and age more opaque. This was demonstrated in a study by Flege (2008) who conducted a Principal Component Analysis after encountering serious multicollinearity issues between the three variables. The analysis revealed that age of onset loaded on a different factor than LoR and age, which loaded on the same factor. This means that the unique contributions of LoR and age at testing remain unclear. These issues have long highlighted the need for more sensitive and reliable measures of exposure than the traditionally established ones. Flege (1991), for example, proposed the Full Time Equivalent measure to estimate quantity of input instead of relying on LoR alone, and more recently Unsworth (2013) proposed an alternative measure of current and cumulative exposure that is more detailed and informative. In response to this issue, we have also derived an alternative measure of input, which is described below.

In the current study, we set out to explore the potential role of two predictors of L1 and L2 grammatical proficiency in L1 Greek-L2 English adult bilinguals: aptitude – more precisely, grammatical sensitivity – and input.

Grammatical proficiency was assessed by means of a grammaticality judgement task (GJT). Despite some criticism on the validity and reliability of GJTs (Platt, 1991; Devitt and Devitt, 2006; Tabatabaei and Dehghani, 2012; Orfitelli and Polinsky, 2017) many researchers still prefer them over other comprehension tasks (Linebarger et al., 1983; Van der Lely et al., 2011). More specifically, GJTs have been extensively used in both L2 (Plonsky et al., 2020) and L1 research (Ambridge, 2012, 2014), and have been employed in a large amount of research concerned with the Critical Period Hypothesis (e.g., Johnson and Newport, 1989; Flege et al., 1999; DeKeyser, 2000; Birdsong and Molis, 2001). This is because, in contrast to other grammar tasks, GJTs can test a wider variety of morphosyntactic structures with different degrees of difficulty and also different levels of association with prescriptive grammar instruction. Another advantage of GJTs is that they can target elements of “decorative” grammar, i.e., those aspects of a grammatical system that have low functional load (e.g.: grammatical morphemes such as agreement markers); these have been shown to be particularly challenging for L2 learners (White, 2003; Hopp, 2010; Dąbrowska et al., 2020).

Language aptitude was tested through the Sentence Pairs task (Llompart and Dąbrowska, in press), which is based on the Words in Sentences subtest of the MLAT and assesses grammatical sensitivity. Finally, as far as input is concerned, we computed a composite measure of exposure which was calculated based on the participants’ years of language use across different periods of their lifetime (for details see Method section). This measure of exposure was used in combination with LoR to test the sensitivity of the one against the other.

In addition, participants completed a Pseudoword Learning task which assessed explicit associative memory, i.e., an aspect of language aptitude which is most relevant for the acquisition of lexical knowledge. Including this task thus allows us to determine whether any effects of aptitude on grammar are component-specific (i.e., only grammatical sensitivity is relevant for grammar) or more general (other aspects of aptitude contribute to grammar).

Furthermore, age and education were included as covariates because it has been shown that they can influence grammatical performance in both L1 and L2 speakers. Dąbrowska (2019) found that age at testing showed a different pattern of correlations in L1 and L2 speakers: in L1 speakers, it was positively correlated with vocabulary and collocations; in L2 speakers, it was negatively correlated with grammar. With regard to education, it is a contributing factor in individual differences among both L1 (Chipere, 2003; Street and Dąbrowska, 2010; Llompart and Dąbrowska, 2020) and L2 speakers (Hakuta et al., 2003; Tarone and Hansen, 2007; Janko et al., 2019). Specifically, speakers with higher academic achievement typically perform better than those with low academic achievement, and the latter group also tends to present a much wider distribution of scores. Finally, education can be quite revealing of the quality of input that the participants have received, especially in the case of participants who started learning the L2 in instructional settings.

## Materials and methods

### Participants

A total of 75 participants (60 females) took part in this study. They were all adults (mean age = 35.9 [23–52], SD = 6.9), who had not been diagnosed with any speech or cognitive disorders. According to self-reports, their mean age of arrival to the UK was 27.3 [17–44] (SD = 6.4), with a mean length of residence of 8.7 [2–30] years (SD = 5.6) and they had on average 18.1 [11–28] years of total education (SD = 2.9). All participants were first generation immigrants.

### Materials

#### Background questionnaire

All participants completed a background questionnaire which contained questions on language history, input, and attitude towards both languages and took around 10 min to complete. In one of the input questions participants were asked to indicate how much they had used English versus Greek in 5 different periods of their life by choosing a value on a five-point scale ranging from ‘All English’ to ‘All Greek’. The periods were as follows: from birth to first exposure to English; from first exposure to English until the end of primary school; secondary school; from end of secondary school until first arrival in the UK; and from first arrival in the UK until the present. This information was used to derive a measure of cumulative exposure to each language. For example, if a participant had said that they spoke ‘Half English/Half Greek’ during secondary school that was transformed into 50% English and 50% Greek. Considering that this period covers 6 years of life we would say that they spent 3 years speaking English and 3 years speaking Greek. The same was done for all the aforementioned periods and the years of use were then added up for each language separately. After that we computed the proportion of Greek use by dividing the years of cumulative exposure to Greek by the sum of the years of cumulative exposure to both Greek and English. This gave us a proportion value, which meant that the higher the number the more the use of Greek and is referred to merely as ‘exposure’ in the current study.

#### Grammaticality judgment task

For the Greek GJT, we selected a number of structures that pose difficulties for either L1 and L2 speakers of Greek (or both). After a pilot study with native speakers, we selected five structures (see Table 1). Three of these (agreement attraction, aspect, and past perfective) were challenging even for native speakers; the remaining two structures (adjective-noun and subject-verb agreement) are relatively easy for native speakers. There were 24 sentences in each category.

**Table 1:** Examples of morphosyntactic structures used in the Greek GJT.

**Structure**	**Example (ungrammatical)**
**Agreement attraction**	*I elipsi trofimon se poles polis tis Venezuelas eχun ftasi se anisiχitika epipeða.
	The lack^-NOM.3SG.SBJ^ food^-GEN.3PL^ in many cities of Venezuela reach^-PRS.PRF.ACT.3PL^ to alarming levels.
	“The lack of food in many cities of Venezuela *have* reached alarming levels.”
**Aspect**	*Eno o Nikos ke i Katerina χorepsan, ksafnika i musiki stamatise.
	While Nick and Catherine dance^-PST.PRF.ACT.3PL^ suddenly the music stop^-PST.PRF.ACT.3PL^.
	“While Nick and Catherine *danced*, (suddenly) the music stopped.”
**Past perfective**	*Tin proiγumeni paraskevi i Katerina estalse ena ðema stin fili tis sti θessaloniki.
	Last Friday Catherine send^-PAST.PERF.ACT.3PL(ungrammatical regularization)^ a package to friend in Thessaloniki.
	“Last Friday Catherine *sended* a package to her friend in Thessaloniki.”
**Adjective-noun**	*O traγikos telos tis tenias prokalese θlipsi stus θeates.
**agreement**	The^-M^ tragic^-M^ end^-N^ of movie cause sadness to spectators.
	“The tragic end of the movie caused sadness to the spectators.”
**Subject-verb**	*To pinasmeno aγori kanun parapona sti mitera tu.
**agreement**	The hungry boy^-3SG.SBJ^ complain^-PRS.ACT.3PL^ to his mother.
	“The hungry boy *are* complaining to his mother.”

For the English GJT, we used a subset of the stimuli developed by Llompart and Dąbrowska (in press). These included five structures which have been shown to be challenging even for native speakers as well as several types of “easy” structures (violations of subject-verb and determiner-noun agreement, negative sentences without do support, and plural and past tense marking errors) on which native speakers perform at ceiling. Examples of each category of item are provided in Table 2. There were 20 sentences for each of the “difficult” items and 20 “easy” items.

**Table 2:** Examples of morphosyntactic structures used in the English GJT.

**Structure**	**Example (ungrammatical)**
**Agreement attraction**	*The structure of the new buildings *are* fascinating.
***That* Trace**	*What do you think *that* will be this year’s revenue?
**Subcategorization**	*My friends and I really enjoy *to play* football.
**Stranded Wh-Questions**	*What does your mum believe *what* your aunt meant by that?
**Double tense**	*When did the last election *took* place in North America?
**‘Easy’ structures**	*Many kids *has* problems with adjusting to high school.

Thus, both the Greek and the English GJT contained 120 items (60 grammatical and 60 ungrammatical). All the sentences were recorded by native speakers of the respective language. The audio files were edited to improve the sound quality and adjust the onset and offset of the stimuli. In both tests, the items were presented in a semi-random order, with the constraint that no more than two items belonging to the same category appeared in a row and no more than three consecutive items belonged to the same response category (i.e., grammatical/ungrammatical). All test items were presented in the exact same order ensuring that any order effects are the same for all participants.

At the beginning of both tasks, participants were provided with detailed instructions which included 2 practice trials (presented in writing) with feedback. Before the test trials participants were asked to click on ‘Play’ to listen to a trial audio to adjust the volume on their device. Every test trial started with a 700 ms fixation cross and then participants could see a “Play” button on their screen and the sentence “Click on Play to listen to the sentence” on top of their screen. At the bottom of the screen, they could also see the two response buttons that they had been instructed to click on based on what they had heard (green tick for correct or red cross for incorrect). The location of the response buttons (correct-on the left and incorrect-on the right) was the same for all participants. After the 60^th^ trial participants were encouraged to take a short break. The total duration of each task was *circa* 15 min.

#### Pseudoword learning task

The Pseudoword Learning task was based on the novel word learning paradigm used in Llompart and Reinisch (2017, 2020, 2021) and was designed to test participants’ abilities to learn word-picture associations. The task included 10 nonce words following English phonotactics and ten pictures of novel objects chosen from a novel object database (Horst and Hout, 2016). The novel words had all been recorded by a native speaker of English. The task was divided in two parts: training and testing. In this study, we only focus on the training phase. During training, participants were presented with a set of four images on the screen. On the upper part of the screen, between the first two images, there was a “Play” button that participants had been instructed to click on to listen to the target word. After playing the audio, they had to choose the item that corresponded to the word they heard by clicking on one of the images. Each target word was presented together with three distractors. Feedback was provided throughout the whole training phase and after each answer (both right and wrong) the correct image appeared on the screen and participants also listened to the object’s name. Training included six blocks with ten words each (=60 trials). The entire task took about 15 min to complete.

#### Sentence pairs task

We used the Sentence Pairs task developed by Llompart and Dąbrowska (in press), which is an adaptation of the Words in Sentences task from the MLAT. The task measures participants’ sensitivity to grammatical structure without using any grammatical terminology (e.g., adjective, object etc.). The task was administered in English. Participants were presented with pairs of sentences. In each pair, the first sentence (also referred to as the key sentence) contained a word printed in capitals. The second sentence contained five words printed in bold. For example, a pair would consist of the key sentence “The laptop was ON the table when I left” and the second sentence “The mouse just started running and hid under the fridge.” Below each of the five words in bold there was a response button. Participants were instructed to press the response button for the word from the second sentence that had the same grammatical function as the word in capitals in the key sentence (under for the example above). Participants were provided with detailed instructions and four practice trials with feedback to familiarize them with the task and ensure that they had understood the instructions. During the practice trials, if participants clicked on the correct response a green tick appeared on the screen whereas if they gave a wrong response then a red cross was displayed. No feedback was provided during the test trials. The total number of test trials was 32 and the task took approximately 15 min to complete.

### Procedure

The participants were recruited online *via* social media (Facebook) or Prolific (an online participant recruitment platform) and received monetary compensation for their contribution. All the tasks were administered online. Before the experiment, participants were provided with detailed information about the tasks and were invited to consent to taking part. If they consented, they were taken to the background questionnaire (see Supplementary material) which was administered *via* the online survey platform (Formsite - online form builder. Create HTML Forms and Surveys [WWW document], 2022). The remaining tasks were administered *via* the Gorilla experimental platform (Anwyl-Irvine et al., 2020). The study was reviewed and approved by the Research Ethics Committees of the Friedrich Alexander University of Erlangen-Nuremberg and the University of Birmingham [ERN_16-0608AP23].

## Results

### Data pre-processing

For both the Greek and English GJT, we excluded response trials in which participants had not clicked on ‘Play’ before responding. That resulted in the exclusion of 43 trials (0.47%) from the complete dataset in the English version and 15 trials in the Greek version (0.16%). The same criterion was applied to the Pseudoword Learning task, which led to the removal of 16 trials (0.35%). No trials were removed from the Sentence Pairs task.

### Descriptive statistics

The descriptive statistics for each task are provided in Table 3. For the GJTs we provide overall accuracy scores and accuracy for grammatical and ungrammatical items separately. In addition to accuracy scores, we computed d-prime scores following Huang and Ferreira (2020). D-prime scores, which are based on Signal Detection Theory, are considered to be a more sensitive measure, but our correlation analysis revealed that percentage and d-primes scores correlated very strongly with each other for both Greek (*r = 0.94*) and English (*r = 0.97*). As a result, we decided to only use percentage scores because they are more easily interpretable.

**Table 3:** Proportions of correct responses, SDs, ranges and IQR for all tasks.

Measures	Mean	SD	Range	Interquartile range
Greek GJT (overall)	0.93	0.04	0.78–0.99	0.91–0.96
grammatical	0.97	0.02	0.86–1.00	0.96–0.98
ungrammatical	0.89	0.07	0.62–1.00	0.83–0.95
English GJT (overall)	0.68	0.10	0.48–0.91	0.62–0.75
grammatical	0.86	0.08	0.61–0.98	0.83–0.93
ungrammatical	0.50	0.17	0.18–0.93	0.39–0.58
Sentence Pairs Task	0.78	0.14	0.28–1.00	0.72–0.88
Pseudoword Learning Task	0.74	0.10	0.45–0.95	0.68–0.82
Exposure (to Greek)	0.75	0.10	0.51–0.95	0.69–0.83

### Correlational analysis

Table 4 shows the pairwise correlations between all variables. The demographic and contextual variables (Age, Education, LoR and Exposure) were extracted from the background questionnaire. Education was based on the total number of years of education in both countries (Greece and United Kingdom) and Exposure (to Greek) was measured by deriving a proportion score (as described in section Background questionnaire).

**Table 4:** Pairwise correlations between all the variables.

	Greek GJT	English GJT	LoR	Exposure	Age	Education	Sentence pairs	Pseudoword task
Greek GJT	1	0.51	0.01	−0.19	0.14	0.17	0.37	0.09
English GJT	0.51	1	0.26	−0.45	−0.1	0.21	0.18	0.04
LoR	0.01	0.26	1	−0.53	0.49	0.15	−0.09	−0.15
Exposure	−0.19	−0.45	−0.53	1	−0.01	−0.17	0.07	0.02
**(to Greek)**								
Age	0.14	−0.1	0.49	−0.01	1	0.12	−0.06	−0.21
Education	0.17	0.21	0.15	−0.17	0.12	1	0.03	0.2
Sentence Pairs	0.37	0.18	−0.09	0.07	−0.06	0.03	1	0.07
Pseudoword Task	0.09	0.04	−0.15	0.02	−0.21	0.2	0.07	1

As can be seen from the table above, there is a strong relationship between the Sentence Pairs task and the Greek GJT (0.37), exposure and the English GJT (−0.45), as well as between the two GJTs (0.51).

### Linear mixed-effects regression analysis

Statistical analysis was conducted in R Core Team (2021). A linear-mixed effects regression model (*lme4* package; Bates et al. (2015) was fit with *Grammar* (i.e., proportion of correct responses on the GJT) as the dependent variable. The independent variables in the model were *Language* (English vs. Greek), *Age*, *Education*, *LoR*, *Exposure*, and individual scores in the *Pseudoword Learning task* and *Sentence Pairs*. All measures obtained from the tasks were entered as the proportion of correct responses by participant, including the dependent variable (*Grammar*), and all variables (both dependent and independent) were standardized using the scale function in R. The variable Language was contrast coded (−0.5 for English and 0.5 for Greek) and was treated as numeric in the analysis. The model also included two-way interactions between Age and Exposure as well as the interactions of Language with three other variables, namely Exposure, Sentence Pairs and LoR. With this analysis, we aimed to examine the potential effects of aptitude (i.e., Sentence Pairs) and input (i.e., self-reported exposure) on L1 and L2 grammatical proficiency, while also accounting for the potential influence of education, age, and explicit associative measure (i.e., Pseudoword Learning task).

A full model containing all our independent variables and two-way interactions as fixed effects and random intercepts by participant was fit first. We then performed model comparisons to obtain the best-fitting model. For these comparisons, we removed predictors one by one, starting with the interaction terms, following the procedure recommended by Crawley (2013). After the removal of each predictor, we performed a log-likelihood ratio test in order to test model fit. If having the predictor in the model improved the fit, it was retained; otherwise, the predictor was removed. This process was repeated until all predictors had been tested. The data and code to reproduce the analyses conducted in this article can be accessed *via* the following link.^2^

The final model, as summarized in Table 5, revealed significant main effects of Language, Exposure and Sentence Pairs, as well as a significant interaction between Language and Exposure. This means that the participants’ performance was better in their native language (Greek) than their second language (English). Furthermore, higher values of Exposure (i.e., a higher proportion of exposure to Greek) and higher scores on the Sentence Pairs task were associated with better performance.

**Table 5:** Coefficients and significance values for the final, best fitting linear mixed effects model.

Predictor	*b*	*t*	*p*
(Intercept)	−0.000	−0.002	0.998
language	1.681	26.710	<0.001***
Exposure (to Greek)	−0.194	−4.399	<0.001***
Sentence Pairs	0.131	2.964	<0.005 **
Language*exposure	0.254	4.030	<0.001***

Asterisks indicate level of statistical significance: *p ≤ 0.05, **p ≤ 0.01, ***p ≤ 0.001.

Following up on the significant interaction between Exposure and Language, we performed least-squared regression analyses with the data split by Language. This was done based on the best-fitting model (see Table 5) such that the two models, one for the Greek data and one for the English data, contained both Sentence Pairs and Exposure as predictors. We tested whether the models could be further simplified as described above but this was not the case. The final models for each language are presented in Tables 6, 7. Along with the coefficients, t-statistics and significance levels, we also reported the lmg metric (i.e., the relative importance of each predictor) as obtained by means of the *relaimpo* package in R (see Groemping, 2007). This measure “expresses the unique contribution of the IV to the total variance of the DV” (Tabachnick and Fidell, 2000, p. 145), thus showing how much each variable contributes to the model’s overall R^2^.

**Table 6:** Final model for the Greek GJT.

Predictor	*b*	*t*	*p*	lmg
(Intercept)	0.911	22.14	<0.001***	
Exposure (to Greek)	−0.093	−2.07	<0.05 *	0.04
Sentence Pairs	0.117	3.64	<0.001***	0.14

Asterisks indicate level of statistical significance: *p ≤ 0.05, **p ≤ 0.01, ***p ≤ 0.001.

**Table 7:** Final model for the English GJT.

Predictor	*b*	*t*	*p*	lmg
(Intercept)	0.914	9.938	<0.001***	
Exposure (to Greek)	−0.454	−4.504	<0.001***	0.20
Sentence Pairs	0.146	2.025	<0.05 *	0.03

As shown in Table 6, Sentence Pairs was the main predictor, accounting for 14% of the variance, whereas Exposure had a much smaller effect and accounted for just 4% of the variance. This means that higher scores in the Sentence Pairs task and, to a smaller extent, lower exposure values (i.e., less Greek, more English), resulted in higher scores in the Greek GJT.

As for English, both Exposure and Sentence Pairs were significant positive predictors of grammatical performance. However, in this case, the lmg analysis revealed that most of the variance in our model was explained by Exposure (20%) whereas the Sentence Pairs task had a much smaller contribution (3%). In other words, less exposure to Greek and more to English was associated with higher scores in the English GJT. The results for English are shown in Table 7.

## Discussion

The aim of this study was to explore learner-internal and environmental factors that contribute to ultimate grammatical attainment in adult Greek-English bilinguals. Our main predictors were aptitude (and specifically, grammatical sensitivity), and exposure. Age, education, and explicit associative memory (as assessed by the Pseudoword Learning task) were treated as control variables. The present results are relevant in at least two major respects. First, there is a positive relationship between aptitude and proficiency in both the participants’ first and second language. Moreover, there is a strong relationship between exposure and grammatical proficiency in the second language. Below we will try to unravel the significance of these findings as well as their implications.

### Effects of input

We explored the role of input by comparing its effect on both first and second language grammatical proficiency. We used two different measures of input: the traditional measure of LoR and a new, more nuanced measure of cumulative exposure across the lifespan. Crucially, when both measures were included in our regression model, only the latter showed a significant effect. This is also evident in the correlation results where we see that the relationship between English grammatical proficiency and cumulative exposure was substantially stronger (−0.45) compared to that between English grammatical proficiency and LoR (0.26). (Note that the correlation with cumulative exposure is negative because our measure is based on the proportion of time the participants used Greek: in other words, a higher value equals to more exposure to Greek compared to English.)

This new measure of input revealed some interesting results. We found that exposure was more strongly related to grammatical performance in the second language. To be more precise, exposure amounted for 20% of the variance in the second language but only 4% in the native language. This does not mean that input does not matter for the native language: rather, as discussed earlier, exposure to the L2 may matter more for the participants at this stage of their life because they have had much less of it (and consequently are still benefiting from it). This is also evident in Table 3, since the mean exposure value reported there is 0.75, which reflects a larger amount of exposure to Greek than English. On the other hand, participants at this age (mean = 35.9) are likely well beyond the stage in which input differences substantially contribute to L1 grammatical proficiency. This fits well with the results reported by Hartshorne et al. (2018), who found that native speakers’ grammatical performance continues to improve until approximately age 30.

One surprising finding was that the effect of exposure on L1 grammar had a negative coefficient in the model, meaning that more exposure to the second language was associated with better performance in the native language -- although it must be noted here that the effect is so small that it may not be very informative. A possible explanation is that learning a second language in adulthood leads to an increase in metalinguistic awareness, which could improve performance on grammatical tasks in the L1 as well.

Overall, our findings highlight the significant contribution of exposure in second language learning and suggest that the magnitude of its effects might even have been underestimated in previous studies due to the unreliable ways of measuring it.

### Effects of aptitude and their specificity

Perhaps the most interesting finding of the paper is the effect of aptitude, more specifically the component of grammatical sensitivity measuring explicit metalinguistic awareness, on grammatical proficiency in the native language. This finding contradicts the claim that aptitude is only relevant for L2 learning and is consistent with recent work reporting correlations between aptitude and native language skills (Skehan and Ducroquet, 1988; Sparks et al., 2009; Dąbrowska, 2018; Llompart and Dąbrowska, in press; Winckel et al., in preparation). In fact, the lmg result in our analysis showed that aptitude explained more of the variance in the native language model (14%) than in the L2 model (3%).

It could be argued that the correlation between aptitude and Greek grammatical proficiency could be attributed to the fact that some of the participants are simply better at taking tests, or perhaps better in all tasks assessing verbal abilities. However, we have solid reasons to believe this is not the case, given that we did not find any relationship between either Sentence Pairs or grammatical proficiency and the Pseudoword Learning Task. If the relationship between aptitude and grammar was indeed due to individual differences in test-taking ability, then one would also expect a correlation between the Pseudoword Learning task and our other measures, which is not the case. In addition, the lack of a relationship between the Pseudoword Learning task, which targeted a component of aptitude that is crucial for the learning of vocabulary, and grammatical proficiency supports the view that the effect of aptitude is not general but component-specific, and possibly restricted to the aptitude components that are known to be relevant for grammar learning.

However, the relationship between aptitude and grammatical proficiency is quite complex and likely non-transparent, and we cannot be fully conclusive with regard to its exact origin. To begin with, one thing that should be interpreted with great caution is the directionality of the causation between aptitude and the L1. There are two main possibilities. The first one is that language learning in general relies on grammatical sensitivity, and this could explain why we find a relationship between aptitude and both L1 and L2 performance. This argument fits in with our findings considering that we found an effect of aptitude for both languages, but of different magnitude. Moreover, the aforementioned point has also been put forward by Llompart and Dąbrowska (in press) who found a similar relationship between aptitude and L1 grammar. The second possibility is that grammatical sensitivity develops through first language acquisition and is a product of L1 development as proposed by Skehan and Ducroquet (1988) and Sparks et al. (2009), hence the relationship we find between aptitude and first language development. Finally, another scenario could be a bidirectional or mutual influence, in which learning a language results in greater grammatical sensitivity and increased grammatical sensitivity leads to more efficient language learning.

It is also important to note that, since our participants were adults, we cannot draw any conclusions about the point in development when the interactions between grammatical sensitivity and grammatical development take place. It is possible that such interactions occur relatively early in development (i.e., in childhood), but they could also have taken place in adolescence or early adulthood. This question could be addressed in future research by conducting longitudinal studies in order to pinpoint the time course of these interactions.

## Conclusion

To sum up, the novelty of this study lies in the fact that it tested the same group of bilingual participants in their first and second language. This provided us with an ideal testing ground for the effects of linguistic and extralinguistic factors on grammatical knowledge. The results challenge the claim that language aptitude is only relevant for L2 learning and suggest that, in fact, it may be even more relevant to L1 acquisition. Last but not least, the current findings provide evidence that our measure of cumulative exposure can better account for individual differences in L2 grammatical proficiency than the measure routinely used in ultimate attainment studies, namely length or residence (LoR).

## Data availability statement

The original contributions presented in the study are included in the article/Supplementary material, further inquiries can be directed to the corresponding author.

## Ethics statement

The studies involving human participants were reviewed and approved by Research Ethics Committees of the Friedrich Alexander University of Erlangen-Nuremberg and the University of Birmingham [ERN_16-0608AP23]. The patients/participants provided their written informed consent to participate in this study.

## Author contributions

All authors designed the study and prepared the experimental tasks. LP collected and processed the data. ED and ML supervised and verified the analytical methods. All authors contributed to drafting and revising the paper and have approved the final version of the manuscript.

## Funding

This project was funded by an Alexander von Humboldt Professorship (ID-1195918) awarded to ED.

## Conflict of interest

The authors declare that the research was conducted in the absence of any commercial or financial relationships that could be construed as a potential conflict of interest.

## Publisher’s note

All claims expressed in this article are solely those of the authors and do not necessarily represent those of their affiliated organizations, or those of the publisher, the editors and the reviewers. Any product that may be evaluated in this article, or claim that may be made by its manufacturer, is not guaranteed or endorsed by the publisher.

## Footnotes

^1^Janacsek et al. (2012) do report that children under 12 performed better on an implicit learning task than older participants. However, this difference was only evident when comparing raw reaction times on low- and high-probability sequences, and not on accuracy or Z-transformed RT measures, so it is not clear if the difference is real. Furthermore, the existence of a statistically significant difference between groups does not entail the existence of categorical differences: in other words, it does not follow that (nearly) all under-12 s have better implicit learning abilities than (nearly) all over-12 s.

^2^ https://osf.io/agpwe/?view_only=1fcd3c54c53b470eaecb07ca9fc179d2
