# Supplementary material for: Aptitude and experience as predictors of grammatical proficiency in adult Greek-English bilinguals
Source: Front Psychol. 2022 Dec 20;13:1062821. doi: 10.3389/fpsyg.2022.1062821 (PMC9808080; doi:10.3389/fpsyg.2022.1062821)
Supplement: Supplementary file 1 [file Data_Sheet_1.docx]

Demographic information:

1. Please specify your gender: Female/Male/Other
2. What is your age?
3. At what age did you arrive in the UK? (If you were born here, write 0)
4. Have you received any schooling in Greece? Yes/No
   If yes, how many years of schooling have you received at each level?

| **Educational level** | **Years of schooling** |
| --- | --- |
| Primary |  |
| Secondary |  |
| Higher education (university level) |  |
| Other (specify): ________________ |  |

Have you received any schooling in the UK or another English-speaking country? Yes/No
If yes, how many years of schooling have you received at each level?

| **Educational level** | **Years of schooling** |
| --- | --- |
| Primary |  |
| Secondary |  |
| Higher education (university level) |  |
| Other (specify): ________________ |  |

Comment(s):

1. What is the highest level of education you have completed?
   -Primary education
   -GCSE or equivalent
   -A-level or equivalent
   -Bachelor’s degree
   -Master’s degree
   -PhD

Comment(s): ______________

1. What is your primary caregivers’ native language? ('Primary caregivers' are people who brought you up. For most people this will be the mother and father, but it could be another person, for example a grandmother or nanny).
   Primary caregiver 1: ____________ Primary caregiver 2 (if applicable): ____________
2. What is the highest level of education that your primary caregivers/parents have completed? (please indicate by ticking ✔ the relevant box below).

| **Education** | **Primary caregiver 1** | **Primary caregiver 2** |
| --- | --- | --- |
| Primary education |  |  |
| GCSE or equivalent |  |  |
| A-levels or equivalent |  |  |
| Bachelor’s degree |  |  |
| Master’s degree |  |  |
| PhD |  |  |

8) Which of the options below best describes the variety of Greek you speak?
-Standard Greek
-Northern Greek dialect
-Southern Greek dialect
-Cretan Greek dialect
-Other (please specify): _______________

9) If you have lived or travelled in countries other than your country of residence for six months or more, then indicate the name of your country, your length of stay (in months), the language you used and the frequency of your use of the language, for each country.

| Never Rarely Sometimes Regularly Often Usually Always | | | |
| --- | --- | --- | --- |
| 1 2 3 4 5 6 7 | | | |
| **Country** | **Length of stay (in months)** | **Language** | **Frequency of use** |
|  |  |  |  |
|  |  |  |  |
|  |  |  |  |
|  |  |  |  |

10) List all the languages that you have studied, from the most proficient to the least proficient and answer the following questions about each language:

| **Language** | **Age of first exposure** | **Years of study** |
| --- | --- | --- |
| English |  |  |
| Greek |  |  |
| Other (specify): ___ |  |  |

11) Have you attended Greek heritage classes while living in the UK? Yes/No

If yes, for how long?

Language proficiency:

12) Rate your proficiency in each of your languages, on the following aspects of language. Rate according to the following scale (please write the number in the table):
Not at all Very poor Poor Functional Good Very good Native-like

1____________2__________3__________4__________5__________6__________7

| **Language** | **Speaking** | **Listening** | **Reading** | **Writing** |
| --- | --- | --- | --- | --- |
| 1. *English* |  |  |  |  |
| 2. *Greek* |  |  |  |  |
| Other (specify):______ |  |  |  |  |

Language use and history:

13) How much did you use English versus Greek during the following stages of your life?

|  | | All English | | Mostly English | | Half English half Greek | | Mostly the Greek | | All Greek | |
| --- | --- | --- | --- | --- | --- | --- | --- | --- | --- | --- | --- |
| Now (current language use) | | 🞏 | | 🞏 | | 🞏 | | 🞏 | | 🞏 | |
| Preschool age (0-5 years old) | 🞏 | | 🞏 | | 🞏 | | 🞏 | | 🞏 | |  |
| Primary School age (6-12) | 🞏 | | 🞏 | | 🞏 | | 🞏 | | 🞏 | |  |
| Secondary School age (13-18) | 🞏 | | 🞏 | | 🞏 | | 🞏 | | 🞏 | |  |
| After secondary school (18+) | 🞏 | | 🞏 | | 🞏 | | 🞏 | | 🞏 | |  |

Comment(s):

14) Please indicate which language(s) you generally use when speaking to the following people:

|  |  | | All English | Mostly English | Half English half Greek | Mostly Greek | All Greek |
| --- | --- | --- | --- | --- | --- | --- | --- |
|  | | Parents | 🞏 | 🞏 | 🞏 | 🞏 | 🞏 |
|  | | Siblings | 🞏 | 🞏 | 🞏 | 🞏 | 🞏 |
|  | | Grandparents | 🞏 | 🞏 | 🞏 | 🞏 | 🞏 |
|  | | Other Relatives | 🞏 | 🞏 | 🞏 | 🞏 | 🞏 |
|  | | Partner/Spouse | 🞏 | 🞏 | 🞏 | 🞏 | 🞏 |
|  | | Friends | 🞏 | 🞏 | 🞏 | 🞏 | 🞏 |
|  | | Colleagues/Classmates | 🞏 | 🞏 | 🞏 | 🞏 | 🞏 |

15) At what age did your parents/primary caregivers arrive in the UK?
Primary caregiver: _____________ Secondary caregiver: _________________

16) Indicate all the languages spoken at home by your parent**/primary caregiver** while you were growing up (from birth until the age of 18) and rate his/her proficiency. Rate according to the following scale (write the number in the table):

Not at all Very poor Poor Functional Good Very good Native-like

1__________2__________3__________4__________5__________6__________7

| **Language** | **Speaking** | **Listening** | **Reading** | **Writing** |
| --- | --- | --- | --- | --- |
| 1. *English* |  |  |  |  |
| 2. *Greek* |  |  |  |  |
| Other (specify):______ |  |  |  |  |

Indicate all the languages spoken at home by your parent/**secondary caregiver** while you were growing up (from birth until the age of 18), and rate his/her proficiency. Rate according to the following scale (write the number in the table):

Not at all Very poor Poor Functional Good Very good Native-like

1__________2__________3__________4__________5__________6__________7

| **Language** | **Speaking** | **Listening** | **Reading** | **Writing** |
| --- | --- | --- | --- | --- |
| 1. *English* |  |  |  |  |
| 2. *Greek* |  |  |  |  |
| Other (specify):______ |  |  |  |  |

Attitude:

17) Below are a few statements regarding your attitude towards Greek and English. Please read them carefully and answer as truthfully as possible based on how you feel. After reading each statement, indicate to what extent you agree or disagree with it.

1. Speaking Greek is an important part of my identity.
2. I feel comfortable when I use Greek.
3. I think it is important to maintain my heritage language (Greek).
4. I think it is important to pass my heritage language to the next generation(s).
5. If I had to give up one of the two languages (Greek or English), I would give up English.
6. Speaking English is an important part of my identity.
7. I feel comfortable when I use English.
8. I think it is important to speak the language of the country I live in (English).
9. It is more important for me that the next generation (s) speak(s) English rather than Greek.
10. If I had to give up one of the two languages (Greek or English), I would give up Greek.
